# Supplee Impression Method*Extract from an article published in the *Dental Summary* for October, 1916.

**Published:** 1916-11

**Authors:** E. R. Kibler


### THE SUPPLEE IMPRESSION METHOD.*

BY E. R. KIBLER, D.D.S.

It is essential that we diagnose our case properly before attempting to take an impression. It is just as important to get a correct diagnosis in prosthetic dentistry as it is in any other operation. We should examine the mouth with the index finger for the hard and soft spots and the movable portion of the soft ridge, the location and strength of the muscular attachments, the space between the tuberosities and the coronoid process.

"If the entire vault and ridges are extremely hard and flat and the muscles attached close to the top of the ridge, it is advisable to make a rubber plate for the patient to wear for at least a year or so, until the action of the rubber causes the tissues to become softer, at which time a metal plate may be made with better results. If the mouth has a tendency to soft ridges, with excess amount of soft tissues in the vault, it is wise to advise the patient to have a metal plate. A temporary gold-lined plate should be made, and have the patient wear it for a year or so to reduce the inflammation before making a metal plate.

"It is well to go into the history of the case in hand before making any promises, for the question of muscle-strain and muscular development will play a prominent part, in view of the fact that we are going to use the muscles to hold the plates in their proper position. If the patient has been chewing for a number of years on a few teeth, with a jaw abnormally closed, or on one side only, or has gone without teeth entirely, we can not expect to open the bite and place the jaws in the correct position, expecting them to be fully efficient and remain the same, until they have fully developed to work in their new relations positions. This development should be ac-

* Extract from an article published in the *Dental Summary* for October, 1916.

complished in stages if we expect to give our patient the comfortable use of the plates during the developmental period. Ignorance on this subject has been the cause of considerable loss in the average dental practice, inasmuch as many dentists have made two or more sets of plates for patients, carrying them through this developmental period before securing permanent results, yet only receiving a fee for one set and ending with a dissatisfied patient. If we had properly diagnosed our case in the first place, and explained to the patient that we would have to regulate the muscles and tissues the same as an orthodontist regulates teeth, we could have received a fee for every denture and at the same time held the patient.

"After we have decided what we are going to do, the next thing is to select our impression tray.

THE REQUIREMENTS OF AN IMPRESSION TRAY.

"First. The tray must fit the vault exactly at the rear and the buccal and labial border of the ridge approximately, with one-eighth of an inch leeway on each side.

"Second. Must be one-eighth of an inch short of the buccal and labial muscular attachments.

"Third. When the ridge is hard in front, exact length of proposed plate or within one-sixteenth of an inch of the vibrating soft plate.

"Fourth. Soft ridge in front, within one-eighth inch or less of the full length of plate-to-be.

"Fifth. If the patient is subject to nausea, the rear edge of the tray must be imbedded sufficiently hard to eliminate vibration of the soft tissues. The tray should be the exact length of the plate-to-be.

"The lower impression tray should be cut short enough so that it ends within one-eighth inch longer than the region of the second molar."

Before proceeding with the impression, we should educate our patient as to what we will expect of him in the new procedure. I generally explain that it is fifty per cent. dentist and fifty per cent. patient. We can prepare the compound and get it into the mouth, and then it is up to the patient to do what we tell him. Get him to go through the facial movements, forward as in whistling, and backward as in laughing. Let him practice with you beforehand and see that he can do this, because you will find if the patient has been without properly constructed dentures for a great number of years, he has lost certain movements of the muscles, and you may have some difficulty in getting him to do as you wish; also, be sure that your patient is sitting upright in the chair and in an unstrained position. You can readily see that if the head is tilted backward and in a strained position it is impossible to give the face movements or close the jaws accurately.

### WATER HEATER.

Mr. Supplee has devised a water heater for heating modeling compound to be used in taking these impressions. I find that this heater is indispensable, and he described the same as follows:

"The apparatus consists of an electric unit suspended in the surface water of a pan. The winding of this electric unit is such that it is capable of creating three distinct heats. By turning it on full, it will bring the water to a boil, and when the surface is boiling, the bottom will still be below 130°.

"By stirring the water in the entire pan up, it will create a uniform temperature of about 160°. By turning the switch off to low, it will maintain the surface to 160° and the bottom will cool off till it is at or below 140°. A gas unit is suspended in the same way, being controlled by a thermostat. Now, what does this mean to us?

"If modeling compound lies in water of not over 150° for ten minutes or more, it will lose some if its stickiness and at the same time work more smoothly.

"The modeling compound most adaptable for our work should be in its best working condition for inserting into the mouth when heated in water 160°.

"Compound can lie in water of 140° temperature for eight to ten hours without spoiling, but it will deteriorate materially if left for an hour in water of 160° or over. If put in boiling water, it will be spoiled in about two minutes.

"Hence we can let our compound lie in the cold water in the bottom of the pan, and when we need it we can reach in with a glass spatula and get the amount we want and mold it in the hot surface-water ready to insert into the mouth.

"Our heating apparatus is of great value as a reducer of temperature, for in many instances we are compelled to heat the compound over a direct flame and to a temperature far beyond the scalding point. Yet by dipping it in the surface water of our pan we will reduce the temperature of the compound so that we can pass it directly into the mouth without the least danger of burning, and with the positive assurance that it is at the right molding temperature."

### SELECTION OF TRAYS.

The trays most advisable for this work are those designed by Mr. Supplee. I mention this because many have purchased other trays through error, thinking they were getting Mr. Supplee's.

These trays have reversible handles conveniently attached under the tray, which can be used as a holder for the biting block as well as a handle.

The Greene trays can also be used, but not being designed for this special technic, are lacking in the essentials.

"The essentials of an upper impression are mouth closed and muscles in the middle position. Compress muscles in relaxed position. Relief of all hard spots.

Equalize pressure on hard and soft tissues and seal back of tuberosities when the mouth is closed.''

HARD RIDGE IN FRONT.

We are now ready for our impression. Dry tray and form compound in a ball about size of a walnut. Hold one side in flame until it flows. Then stick this portion on tray and spread so that the heated part is next to tray. Hold in the hand and place each thumb on each side of the ball of compound, and press downward from the center. Throw up rim around the tray to the region of the first molar. Then pinch up a little mound of compound in the center, leaving the rear of the vault free. Heat this little mound over the flame, allowing the flame to glance off the mound, and heat the sides and rims of the compound. Do this slowly, and allow the heat to penetrate. Dip entire impression material (but do not get the tray in the water) in hot water at a temperature of 160°. This will temper the compound and will not burn the patient. With the left arm around the head of the patient, hold lip up, insert impression sidewise and rotate same, and center over ridge, push impression upward and backward with index finger in the rear third. Press up to place with a wave-like motion, having the patient give the facial movements. Be sure and have the patient give the facial movements while you are gently pushing to place. Do not push to place and then have the patient give the facial movements. Allow to cool for two minutes. If you have an assistant, allow him to throw ice water in the mouth with a Spooner quick-filling syringe. This basic impression is very important, and at this stage we should take our time and allow to thoroughly cool before removing. Remove, trim to proper thickness. Bend away tray in rear and return to mouth and test for rocking by lateral pressure. First on one side in the bicuspid region and then the other. If the impression does not rock, you are ready for the next stage. If the basis impression rocks, you want to run your water heater to 170°, and with your Spooner syringe flow the hot water over the impression, allowing the water to run off the heel. Return to mouth, press to place and cool. If the rock is not out, repeat operation. As a rule you will correct the rock by performing this operation twice. Dry off the impression tray, turn handles out. Take roll of compound and lay on the back of the impression tray. Bring your handles around and tuck compound under them, which will help to hold it. Shape up with fingers; pass compound over the flame; dip in the water and return to mouth. With the occlusal-plane paddle, insert in the mouth and bring up until the handle of the paddle is parallel with an imaginary line drawn from the lower border of the external auditory meatus to the ala of the nose. Remove from the mouth, take paddle off the occlusal plane; this represents the plane which is the equivalent to the position the tips of the occluding teeth assume when the mouth is closed normally. Dip in your ice water and trim up the excess. Return to mouth. If this operation is correctly done, it will give you the correct length of your teeth and the occlusal plane. With upper impression in the mouth, allow the patient to bring the lips together and swallow and hold. Then pull down the lower lip and note the distance between the occlusal plane and the ridge below. This will give you an idea of how much compound to use for your lower impression. We are now ready for the lower impression.

Select the tray; adjust the handle. Be sure the impression tray is dry and short enough; should extend slightly beyond the second molar. The water at this stage should be 170°. Roll the approximate amount of material between the two hands until it is the length of the plate-to-be. Then dip into cold water quickly and dry. This will chill the surface. Then bend to the form of the tray. Pass the surface of compound that is to go next to the tray through flame until it flows, then quickly stick it all around to the inside of the tray. Then dip tray in cold water. This will chill the surface of the compound next to the tray and will prevent compound from flowing over the occlusal plane, also will give you three degrees of temperature in compound. That next to the tray will be hard, the middle will be medium soft, and the portion that will come in contact with the lower ridge will be the temperature of the hot water. Then reverse and hold the face of your compound in hot water until it is just soft enough that you can work out in approximate shape for proposed impression. Be sure that the impression is shaped so that it will strike the sides of the ridge first and flow toward the center, and that you have more material in the region of the ten fronts than in the molar regions. If you have more compound in the molar regions than you have in the incisor region, it will cause the patient to throw the jaw forward. Pass the impression over the flame and flow compound; then hold in the surface of the hot water to equalize heat; then step in front of the patient with the upper impression in place, and quickly pass lower impression into the mouth. Pull the impression forward and allow it to rest on the inside of the lower lip; then ask the patient to bring the lips together and swallow. That will automatically take the impression and give you the bite at the same time. Allow to harden for three minutes; then with your water syringe flood the mouth with ice water, with your saliva ejector drawing the water out. Be sure that your impression is absolutely hard, so that you will not distort impression in removing. Remove from mouth, dip in cold water and allow to remain a few seconds, then take out and trim the impression. Return to mouth and test impression by trying to rock it with pressure one side then the other, in the bicuspid region. If the impression does not rock and does not bob up and down while the patient opens and closes the mouth, you are ready to take the next step.

At this time you should find out whether we have a true bite or not. The way to find this out is to have the patient close the jaws quickly with a snap and listen to the sound, whether it is clean-cut or whether there are two sounds, a strike and a slide. If you decide there is a strike and a slide, warm the occlusal plane of the upper all around one-eighth of an inch deep and return to mouth with the lower in place. Have the patient bring the lips together and swallow. If the occlusal plane has passed through more material on one side than the other, this proves that the patient is biting under muscle strain. In other words, the mandible is tilted slightly or the base-plates have been shifted, due to unequal pressure over the hard and soft areas in the mouth. What we want to do now is to straighten it up. Cut the occlusal plane until it is flat. In other words, remove all indentations. Then cut down the side that passes through the most material, the thickness of a cardboard or more; then warm the entire surface again and return to mouth and have patient bring lips together and swallow. The chances now are, that the occlusal plane of the lower will pass through the same amount of material in the upper plane. If you should close the jaws too much in correcting the bite, take a small roll of compound and put over the occlusal plane of the upper, and return impression to the mouth, having the patient bring the lips together and swallow. This will open your bite again. We are now ready to proceed with our impressions. We are going to take these impressions under biting stress.

We will now adjust our lower impression in the masseter region. Cut away the impression in the region of the molars. Cut off all surplus that has been drawn up by the action of the cheek and tongue, and reduce it to the approximate fullness of the plate-to-be, as far as the cheek and tongue are concerned. Now cut off the impression at buccal and labial side in the molar region as far forward as the second bicuspid, being very careful to cut entirely too much rather than too little. Pass both surfaces that have been cut over the flame to gloss the same, then quickly take a pinch of soft compound from heater and lay it on the ends that have been cut away and shape same to fit the ridge. Hold over the flame and flow same, flowing compound to where you added it. Dip in the hot water and return to mouth. Locate the impression over the ridge and press down gently into place in the region of the ten fronts; then ask the patient to bring the lips together and swallow and close firmly, giving the face movements.

If you had enough compound, you have now conformed your impression to the masseter muscles in their various working positions under biting stress. When the compound has passed from the flowing stage to the molding stage you should gently pull the cheeks upward so as to relax same, if they have been overstrained. Allow to cool, then flood mouth with ice water. When the impression is hard you can test the same for rocking. If it does rock and will not stay down when the patient talks, you should warm the lingual surface of the impression in the molar and bicuspid regions and return to mouth, having the patient bring the lips together and swallow. The lingual attachments, after having been overstrained, will turn up your compound and give you an unstrained impression of the lingual attachments. It is very readily seen that we can overstrain our muscles and tissues by having our compound too hard, therefore it is essential that we have our compound in a flowing state when taking these impressions. We are now ready to proceed to finish the upper.

### FINISHING THE UPPER.

Cut down the buccal and labial rim of our basic impression until it is entirely too short. Then hold over the tip of the flame and gloss the surface that has been cut; then pick up a small piece of compound and roll between the fingers and dip in the water again to warm it; then pass the roll over the flame and lay on the impression where we cut away the rim and pinch too high; then heat this portion over the tip of the flame, allowing to strike on the inside; then dip the entire front portion into the hot water and insert in the mouth. Locate it over the ridge and make sure that the impression is practically seated in front, and then let the patient bite the impression into place; then hold the jaw firm while giving the face movements; then after compound has set for one minute, give a slight massage forward and inward very gently, being sure that you do not do this until the compound has passed from the flowing stage to the molding stage, because if you do this procedure when the compound is flowing, you will cause the same to squeeze up on the buccal and labial attachments and cause a temporary fit. The reason you want to do this is that the forward and backward movements have caused the compound to be pushed out, and the idea is simply to mold it back gently to place against the attachments. In following these steps we have muscle-trimmed the impression so that the rim is the proper height, and made it possible for the muscles to relax. In other words, we have compressed these muscles in their relaxed position, and have done all this with the mouth closed and under biting stress. You should test the plate for rocking from side to side. If your impression should rock at this stage, you should correct it as I told you when taking the basic impression. We will now correct the rear third of the vault.

### CORRECTING THE REAR THIRD.

First examine the vault and refresh in your memory the condition of the tissues and how far forward they extend. We know that they were all stretched out of their normal position when we took the basic impression with the mouth open, so we must now correct same with the mouth closed. That is the reason that a denture will not fit properly when made from an impression with the mouth open. We must cause this material that we are going to add in the rear third of the vault to flow toward the rear, so that it will carry this soft and movable tissue with it, and not confine it under the plate, so that when the mouth is open it will draw the tissue out from under the plate, and then when it is closed will relax same, causing it to try and get under the plate again, therefore producing an uncomfortableness and many times causing the plate to be dislodged.

Take your tracing stick and trace compound across the rear of vault, so as to more than fill up the space that we know exists there. Be sure to add it too far forward and very lightly, directly across the rear edge; then hold over flame and warm entire rear of the impression. Be sure to warm forward of where we added the compound, allowing the flame to glance toward the rear, and be careful not to warm the forward part of the impression. When we have this rear part of the impression in a flowing state, dip in hot water and pass into the mouth, locate over the ridge and press the impression up against the ridge in front, without pressing it up in the back. With the index finger of the left hand against the face of the biting-block, in the region of the medium line pressing backward, ask the patient to bring the lips together, close firmly, and swallow, giving the facial movements, and hold for about a minute; then chill with cold water. Our impression then should stand all tests. The patient should not be able to dislodge with any facial movements. We should not be able to dislodge by pressure with the fingers in any angle. If the impression stands these tests, we can consider the impression perfect. If both impressions test out properly, we have the patient bring the lips together and swallow; that will give us our correct relations of the occlusal plane; then we are ready to put on the face-piece. Do not proceed with the dentures until the impressions stand the above tests.

Take a roll of wax and lay across the face of both upper and lower impressions. Have the patient give the face movements. This will evenly distribute the compound over the impression. Allow to cool, then mark our median line, our smiling line, our lip line, and the impressions are ready to take out of the mouth for the articulator.

### SOFT RIDGE IN FRONT.

The difference between the technic in a hard and soft ridge is as follows:

After we have secured our basic impression, which we allow to run back one-eighth to one-half inch on the vibrating soft tissue (the distance depends upon the softness of a ridge), to compensate for the pressure brought to bear when incising to prevent tipping, we proceed the same as in a hard ridge until we come to the step of replacing our displaced tissue and getting the labial and buccal borders under biting stress. Cut off the labial rim as far back as the soft tissue is displaced. Scrape away the impression under the displaced tissue until you can push the impression to place without displacing the soft ridge; then soften the impression that has been cut and scraped away, by passing through the tip of the flame. Take a small roll of soft compound and lay on a new rim and pass through the flame. Warm all compound that will come in contact with the soft ridge. Dip in hot water, pass into the mouth and have patient close on same; then quickly push warm compound up under lip, ask patient to give facial movements two or three times; then give light massage by pressing gently inward and downward on the lip and cheeks. This will push the displaced tissue back to the normal position. If we allow this tissue to be displaced and make the denture accordingly, we will frequently have pain in the region of the antro-palatine canal, due to the circulation being impaired by the tissue being drawn abnormally forward over the canal. We will also have the patient complain of the plate sagging or not fitting perfectly back of the bicuspids, due to the displaced tissue trying to get back to normal.

